# Teladorsagia Circumcincta Galectin-Mucosal Interactome in Sheep

**DOI:** 10.3390/vetsci8100216

**Published:** 2021-10-04

**Authors:** Nur Nasuha Hafidi, Jaclyn Swan, Pierre Faou, Rohan Lowe, Harinda Rajapaksha, Callum Cairns, Michael Stear, Travis Beddoe

**Affiliations:** 1 Department of Animal, Plant and Soil Science and Centre for AgriBioscience (AgriBio), La Trobe University, Bundoora 3086, Australia; N.Hafidi@latrobe.edu.au (N.N.H.); j.swan@latrobe.edu.au (J.S.); c.cairns@latrobe.edu.au (C.C.); 2 Centre for Livestock Interactions with Pathogens (CLiP), La Trobe University, Melbourne 3086, Australia; 3 Department of Biochemistry & Genetics, La Trobe Institute for Molecular Science La Trobe University, Melbourne 3086, Australia; P.Faou@latrobe.edu.au (P.F.); R.Lowe@latrobe.edu.au (R.L.); K.Rajapaksha@latrobe.edu.au (H.R.)

**Keywords:** galectin, parasite-host interaction, proteomics, *Teladorsagia circumcincta*

## Abstract

*Teladorsagia circumcincta* is the most important gastrointestinal parasite in the livestock industry in temperate regions around the world, causing great economic losses. The infective third-stage larvae (L3) of *Teladorsagia circumcincta* secrete a large number of excretory-secretory (E/S) molecules, some of which are likely to play critical roles in modulating the host immune response. One of the most abundant E/S molecules is a protein termed Tci-gal-1, which has similarity to mammalian galectins. Galectins are a family of carbohydrate-binding molecules, with characteristic domain organisation and affinity for β-galactosids that mediate a variety of important cellular functions including inflammation and immune responses. To understand the role of Tci-gal-1 at the host–parasite interface, we used a proteomics pull-down approach to identify Tc-gal-1 interacting proteins from sheep abomasal scrapes and whole tissue. A total of 135 unique proteins were identified from whole abomasal tissue samples, while 89 proteins were isolated from abomasal scrape samples. Of these proteins, 63 were present in both samples. Many of the host proteins identified, such as trefoil factors and mucin-like proteins, play critical roles in the host response. The identification of Tci-gal-1 binding partners provides new insights on host–parasite interactions and could lead to the development of new control strategies.

## 1. Introduction

Gastrointestinal (GI) nematodes cause significant production and economic losses to livestock industries worldwide [1,2]. Infection with GI nematodes result in significant production losses estimated at approximately 500 million AUD in Australia and 38 million Euro in Europe every year [3,4], while anthelmintics alone are estimated to cost tens of billions of dollars annually worldwide [1,5]. The current control methods are becoming less effective due to the rapid emergence of anthelmintic resistance [6,7,8]. Sustainable control solutions such as vaccines or breeding for enhanced resistance are urgently needed. Increased knowledge of the host–parasite interface would facilitate both approaches.

Of particular importance to the sheep industry is the GI nematode *Teladorsagia circumcincta*, the most significant cause of ovine parasitic gastroenteritis in temperate regions around the world [9]. Infection with *T. circumcincta* elicits a T helper type 2 (Th2) immune response and stimulates the expression of defence mechanisms, including cytokines, immunoglobulins and eosinophils [6,10]. The establishment of infection may cause clinical signs such as weight loss, reduced appetite, profuse scouring, and occasionally, death [11].

The modulation of the host immune response by *T. circumcincta* allows the parasite to persist for long periods of time within the ruminant host [12,13]. This is achieved through the stage-specific release of excretory/secretory (E/S) proteins such as proteases, venom allergen-like proteins, lectins, and other enzymes by the parasite to maintain a state of active suppression [12,14]. These E/S proteins are fundamental for parasite survival in the host, playing a role in host tissue penetration and the feeding and evasion of the host’s anti-parasitic immune response [15]. Each species of helminth secretes unique immunomodulatory molecules capable of targeting different aspects of the host immune response, establishing favourable conditions for parasite survival [16]. Disrupting the activity of these E/S proteins may lead to a quicker elimination of the parasite in a host [14]. An earlier in vitro proteomic study on *T. circumcincta* E/S products revealed that infective L3 and L4 larval stages of *T. circumcincta* produce high levels of galectin [14].

Galectins are a family of β-galactoside-binding lectins that bind to glycans, such as lactose [17,18,19,20]. Galectins are characterised by the presence of at least one carbohydrate recognition domain (CRD) consisting of approximately 135 amino acid residues [21]. This family of proteins can be categorised into three groups: prototype galectins (single CRD), tandem repeat-type galectins (two CRDs with different glycan specificities) and chimera-type galectins (single CRD connected to a non-lectin amino-terminal region) [18,22,23]. Previous studies have demonstrated the differing, and occasionally opposing, roles of galectin inside and outside the cell [24]. Galectins have been implicated in a variety of biological and immune processes such as host–pathogen recognition, cell adhesion, cell growth regulation, T cell polarisation and apoptosis [25].

Genes encoding tandem repeat-type galectins have been isolated from the ovine parasitic gastrointestinal nematodes *T. circumcincta* (Tci-gal-1 and Tci-gal-2)*, Haemonchus contortus* (Hco-gal-1) and *Trichostrongylus colubriformis* (Tco-gal-2) [17], and have been hypothesised to be involved in parasite invasion and immunomodulation [22].

Tci-gal-1 is one of the most abundant E/S proteins secreted upon infection with a largely unknown function [14]. There is limited knowledge about this parasite galectin and the effects it could mediate within the infected host. This study is the first to explore Tci-gal-1-binding molecules by using recombinant Tci-gal-1 to identify galectin glycoconjugates within the host abomasum in order to identify sheep glycoproteins in an endeavour to better understand host–parasite interactions.

## 2. Materials and Methods

### 2.1. Sheep Tissue 

Three sheep abomasa collected from a local abattoir were cut along the greater curvature and thoroughly rinsed with MilliQ water to remove the contents. Whole cuts of tissue from the folds of each abomasum were taken, and mucosal scrapes were also prepared by gently scraping the surface of each abomasum using a microscope slide. The abomasal whole tissue (WT) and scrape (ST) samples were then washed with phosphate-buffered saline (PBS) (137 mM NaCl, 2.7 mM KCl, 1.8 mM KH_2_PO_4_, 10 mM Na_2_HPO_4_, pH 7.4), and 1 g aliquots of each sample were set up in triplicate in 2 mL microfuge tubes and frozen at −80 °C for 20 min. Two 3 mm glass beads (Qiagen, Hilden, Germany) were added to each tube and the samples were homogenised using the Qiagen TissueLyser II (Qiagen, Hilden, Germany) at 30 Hz for two rounds of 5 min. The homogenised tissue was resuspended in an equal volume of 1% (*w*/*v*) sodium deoxycholate (DOC) dissolved in PBS and centrifuged at 16,000× *g* for 20 min. The supernatant was collected and stored at −80 °C until required.

### 2.2. Tci-gal-1 Expression and Purification 

The Tci-gal-1 (NCBI accession number: U67147.1, https://www.ncbi.nlm.nih.gov/ (accessed on 20 March 2019)) gene was commercially synthesised and cloned into the pPICZα vector (Invitrogen, Carlsbad, CA, USA) by Bioneer Pacific (Daejeon, South Korea) via the *Pst* I and *Not* I restriction enzyme sites. The subsequent pPICZα-Tci-gal-1 plasmid translated an N-terminal alpha-factor signal sequence and a C-terminal hexahistidine tag flanking the *Pichia pastoris* codon optimised Tci-gal-1 gene. The pPICZα-Tci-gal-1 plasmid was linearised with *Sac* I digest (New England BioLabs Inc., Ipswich, MA, USA) and chemically transformed into *P. pastoris* X33 using the *Pichia* EasyComp™ Transformation Kit according to the manufacturer’s instructions (Invitrogen, Carlsbad, CA, USA). The transformants were plated onto YEPD agar plates (1% (*w*/*v*) yeast extract, 2% (*w*/*v*) peptone, 2% (*w*/*v*) dextrose, 2% (*w*/*v*) agar) containing Zeocin^TM^ (100 µg/mL) and incubated at 28 °C for 4 days. Single colonies were grown in 10 mL of YEPD broth to make a glycerol stock.

A starter culture of Tci-gal-1 *P. pastoris* cells was grown from a glycerol stock inoculated into 50 mL conical tubes containing 10 mL of YEPD and incubated at 28 °C for 48 h in a shaking incubator (180 rpm) (NB-205LF, N-BIOTEK, Bucheon, Korea). The starter culture was used at a 1:40 ratio to inoculate 400 mL of fresh buffered methanol-complex (BMMY) medium (1% (*w*/*v*) yeast extract, 2% (*w*/*v*) peptone, 1% (*w*/*v*) yeast nitrogen base, 100 mM potassium phosphate pH 6.0 and 0.5% (*v*/*v*) methanol) in a 2 L baffled flask. The cultures were incubated for 96 h at 28 °C whilst shaking (160 rpm), with 0.5% (*v*/*v*) methanol added every 24 h. Cells were pelleted at 6000× *g* for 30 min and the supernatant was dialysed using membrane tubing with a 12 kDa molecular weight cut-off into starter buffer (5 mM NaH_2_PO_4_ pH 7.6, 50 mM NaCl and 2 mM imidazole) at 4 °C for 48 h. Dialysis incubations were repeated at least three times with a minimum of 4 h between each exchange. The dialysed media were concentrated with the application of polyethylene glycol (PEG) 8000 (Astral Scientific, New South Wales, Australia) to the outside of the tubing and kept overnight at 4 °C. 

His-tagged Tci-gal-1 was purified from dialysed supernatant using nickel-nitrilotriacetic acid (Ni-NTA) affinity chromatography. Briefly, 2 mL of Ni-NTA agarose resin (His60 Ni Superflow Resin, Takara Bio Inc., Shiga, Japan) was added to a purification column and equilibrated with 10 bed volumes of starter buffer. Concentrated culture supernatants were added to the column and allowed to flow through by gravity at a rate of 1 mL/min. The resin was then washed with 2 column volumes of wash buffer (starter buffer containing 20 mM imidazole). Bound proteins were eluted with 10 mL of elution buffer (starter buffer containing 250 mM imidazole), followed by 5 mL of elution buffer containing 500 mM imidazole. The elution fractions were pooled and buffer-exchanged using Amicon^®^ 3K Ultra-15 centrifugal filter units into storage buffer (25 mM NaH_2_PO_4_ and 250 mM NaCl, pH 7.6) and stored at 4 °C. Successful expression and purification of recombinant Tci-gal-1 was determined by 12% (*w*/*v*) SDS-PAGE and Coomassie brilliant blue R staining. Protein folding evaluation and identification was performed by circular dichroism spectroscopy [26] and mass spectrometry, respectively (Supplementary Figures S1 and S2).

### 2.3. Determination of Lactose Binding Affinity

The sugar binding affinity of Tci-gal-1 was determined using an assay similar to that described by Greenhalgh and Newton (1999). Briefly, 250 µg of Tci-gal-1 was added to 25 µL of lactose-Sepharose resin (α-Lactose-Agarose, Sigma-Aldrich, St. Louis, MO, USA) equilibrated with PBS containing 4 mM β-mercaptoethanol (MePBS). The suspension was incubated on a rotating wheel at room temperature for 1 h and washed twice with 500 µL MePBS before eluting with 300 µL PBS containing 500 mM lactose. The elution was separated by 12% (*w*/*v*) SDS-PAGE and visualised using Coomassie brilliant blue R staining. 

### 2.4. Conjugation of Tci-gal-1 Onto N-Hydroxysuccinamide (NHS)-Activated Sepharose

Active Tci-gal-1 was conjugated to NHS-activated Sepharose beads (GE Healthcare Life Science, Pittsburgh, PA, USA) as modified from Swan et al. (2019). Briefly, Tci-gal-1 (21 mg) was added to 8 mL of NHS-activated Sepharose beads and allowed to couple on a rotating wheel for 16 h at 4 °C, followed by 2 h at room temperature. The remaining active sites on the resin was blocked for 2 h at room temperature with 100 mM Tris-HCl pH 8.5, 500 mM NaCl and 10 mM tris-2-carboxyethyl-phosphine (TCEP). Then, the resin was washed three times for 10 min at room temperature with 10 volumes of two alternating wash buffers. Wash buffer 1 contained 100 mM Tris-HCl pH 8.8, 500 mM NaCl and 10 mM TCEP. Wash buffer 2 contained 100 mM HEPES-HCl pH 6.8, 500 mM NaCl and 10 mM TCEP. Galectin-conjugated Sepharose was stored in 100 mM Tris-HCl pH 8.0, 150 mM NaCl and 0.05% (*w*/*v*) sodium azide at 4 °C.

### 2.5. Sodium Periodate Treatment 

An aliquot of each lysate preparation was treated with 20 mM sodium periodate dissolved in PBS and 50 mM sodium acetate buffer (pH 4.5), as described by Schallig and van Leeuwen (1996). Glycan modification by periodate treatment was confirmed by conducting a lectin blot. Briefly, 20 µL of 500 µg/mL of periodate treated and untreated whole tissue and scrape tissue were dotted onto a polyvinylidene difluoride (PVDF) membrane and probed with horseradish peroxidase conjugated Concanavalin A (ConA-HRP) lectin (Sigma-Aldrich, St. Louis, MO, USA).

### 2.6. Isolation of Tci-gal-1-Binding Ligands from Sheep Abomasal Tissue

A batch-binding technique was used to capture Tci-gal-1-binding ligands from WT and ST lysates as well as sodium periodate treated WT and ST lysates (*n* = 3). The pull-down experiment was repeated three times from independent prepared lysates from different abomasal tissue.

Approximately 500 µg of each lysate was incubated with 400 µg of galectin conjugated Sepharose beads at room temperature for 3 h on a rotating wheel. Unbound proteins in the supernatant were removed by centrifugation at 500 x *g* for 1 min, followed by three washes with 600 µL RIPA dialysis buffer (20 mM Tris-HCl pH 8.0, 150 mM NaCl, 0.5 mM ethylenediaminetetraacetic acid (EDTA), 0.05% (*v*/*v*) Nonidet P−40, 0.01% (*w*/*v*) DOC and 1% (*v*/*v*) Triton X−100) for 10 min each. Captured ligands were eluted with 600 µL of elution buffer (250 mM lactose, 20 mM Tris-HCl pH 8.0 and 100 mM NaCl) for 30 min at room temperature. The washed and eluted proteins were analysed by 12% (*w*/*v*) SDS-PAGE and visualised by silver staining. 

Briefly, protein bands on the gels were fixed with 40% (*v/v*) methanol and 13.5% (*v*/*v*) formalin for 10 min immediately after electrophoresis. The gels were then washed twice in MilliQ water for 5 min each and soaked in 0.02% (*w*/*v*) sodium thiosulfate for 1 min. After rinsing again with water, the gels were soaked for 10 min in 0.01% (*w*/*v*) silver nitrate and then in developing solution (3% (*w*/*v*) sodium carbonate, 0.05% (*v*/*v*) formalin and 1.6 × 10^−5^% (*v*/*v*) sodium thiosulfate) until protein bands were adequately intensified. The reaction was stopped with 2.3 M citric acid.

### 2.7. Mass Spectrometry

Once the successful isolation of Tci-gal-1 glycoconjugates was confirmed, the eluted ligands were assessed using mass spectrometry (La Trobe University—Comprehensive Proteomics Platform (LTU-CPP), La Trobe University, Melbourne, Victoria). Eluted proteins were precipitated after adjustment to 0.02% (*w*/*v*) deoxycholate and 25% (*v*/*v*) trichloroacetic acid. The precipitated protein was washed in cold acetone before reconstitution in urea (8 M urea, 25 mM Tris-HCl, pH 8.0). Disulphide bonds were reduced by the addition of TCEP to 2 mM for 60 min, followed by the addition of iodoacetamide to 38 mM for 45 min in the dark to alkylate the reduced thiols. The sample was then diluted with 20 mM Tris-HCl to reduce the urea concentration below 1 M before the addition of sequencing grade trypsin (Promega, Madison, WI, USA) to achieve a 1:50 ratio compared to the original protein amount. Trypsin digestion was completed overnight at 37 °C. Tryptic peptides were desalted and concentrated using StageTips according to Rappsilber et al. (2007).

Peptides were reconstituted in 0.1% (*v*/*v*) trifluoroacetic acid (TFA) and 2% (*v*/*v*) acetonitrile (ACN), and 500 ng of peptides were loaded onto C_18_ PepMap 100 µm ID × 2 cm trapping column (Thermo-Fisher Scientific, Waltham, MA, USA) at 5 µL/min for 6 min and washed for 6 min before switching the pre-column in line with the analytical column (Acquity BHE C_18_, 1.7 µm, 130 Å and 75 µm ID × 25 cm, Waters). The separation of peptides was performed at 250 nl/min using a linear ACN gradient of buffer A (0.1% (*v*/*v*) formic acid, 2% (*v*/*v*) ACN) and buffer B (0.1% (*v*/*v*) formic acid, 80% (*v*/*v*) ACN), starting at 5% buffer B to 35% over 90 min, then 50% B in 15 min followed by 95% B in 5 min. The column was then cleaned for 5 min at 95% B following a 5 min equilibration step. Data were collected on a Thermo Orbitrap Eclipse (Thermo-Fisher Scientific, Waltham, MA, USA) in Data Dependent Acquisition mode using *m*/*z* 350–1500 as MS scan range and HCD MS/MS spectra were collected in the orbitrap using a cycle time of 3 s per MS scan at 30,000 resolution. Dynamic exclusion parameters were set as follows: exclude isotope on, duration 60 s and using the peptide monoisotopic peak determination mode. Other instrument parameters for the instrument were: MS scan at 120,000 resolution, injection time Auto, AGC target Standard, HCD collision energy 30%, injection time Auto with AGT target at Standard. The isolation window of the quadrupole for the precursor was 1.6 *m*/*z*. 

### 2.8. Protein Identification and Quantification 

Raw files consisting of high-resolution MS/MS spectra were processed with PEAKS Studio 10 (build) software program (Bioinformatics Solutions Inc., Waterloo, ON, Canada) [27]. Data were searched against an *Ovis aries* database (UniProt, June 2020). Additionally, the spectra was independently searched against the common contaminants database (common Repository of Adventitious Proteins (cRAP)—https://www.thegpm.org/crap/ (accessed on 10 February 2020)). Briefly, signature MS/MS spectra were searched using PEAKS DB algorithms using the carbamidomethylation of cysteine as a fixed modification, and methionine oxidation as well as protein N-termini acetylation set as a variable modification with up to three modifications allowed per peptide. The maximum number of missed cleavages by trypsin digestion was set to two. Mass tolerances were set to ±10 ppm for parent ions and ± 0.5 Da for fragment ions. The minimum peptide length was set to 7, with a maximum mass of 4600 Da. The minimum and maximum peptide length for unspecific cleavage were set at 8 and 25 amino acids, respectively. Protein and peptide spectrum matches (PSM) were reported at a false discovery rate (FDR) of 0.01%. A label free quantification (LFQ) of the proteins was achieved using Andromeda, a built-in search engine within MaxQuant [28]. The label free quantification was done with ‘Match between runs’ using a match window of 0.7 min. Large LFQ ratios were stabilised to reduce the sensitivity for outliers, and data was normalised using the ‘Quantile’ method. Redundant proteins were identified and removed from the raw mass spectrometry dataset, including captured proteins that were present in the negative controls, the cRAP database, as well as proteins that appeared in only one replicate. The average LFQ intensity of each remaining predicted protein was calculated and used to compare protein abundances.

### 2.9. Protein Annotation and Glycosylation Analysis

Unique proteins were identified by searching the accession number of each protein for matches within the Universal Protein Resource Knowledgebase (UniProtKB) consortium database (https://www.uniprot.org/) (accessed on 15 March 2020). The FASTA format protein sequences of uncharacterised proteins were scanned for conserved motifs against the InterPro 76.0 protein signature databases (http://www.ebi.ac.uk/interpro/) (accessed on 15 March 2020), using the InterProScan tool. Additionally, the translated Basic Local Alignment Search Tool (tBLASTn) was used to search for regions of similarity of uncharacterised proteins between sequences in the National Center for Biotechnology Information (NCBI, USA) database. The functional annotations and cellular locations of each protein were predicted by assessing the domains and gene ontology (GO) terms of homologous proteins within UniProt and InterPro. The number of transmembrane domains and signal peptides were noted. Potential N- and O-glycosylation sites were predicted using the NetNGlyc 1.0 Server (http://www.cbs.dtu.dk/services/NetNGlyc/) (accessed on 20 April 2020) and NetOGlyc 4.0 Server (http://www.cbs.dtu.dk/services/NetOGlyc/) (accessed on 20 April 2020) respectively. 

## 3. Results

### 3.1. Expression and Purification of Tci-gal-1

**Figure 1:**
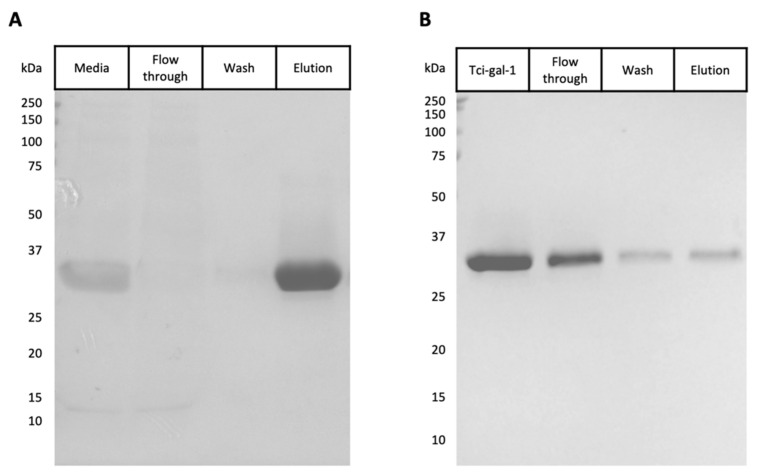
Purification and characterisation of *Teladorsagia circumcincta* galectin (Tci-gal-1)**.** (**A**) *Pichia pastoris* media expressing Tci-gal-1 were applied to a NI-IDA column and washed once before elution with imidazole. A total of 15 µL of each stage of the purification was resolved by SDS-PAGE and stained with Coomassie blue. (**B**) Purified recombinant Tci-gal-1 was shown to be functional by elution from a lactose-Sepharose column with lactose. Equal volumes (15 µL) of each sample were analyzed by SDS-PAGE and visualised with Coomassie blue staining.

Recombinant Tci-gal-1 was expressed in *P. pastoris* and purified by immobilised metal-ion affinity chromatography. The purified protein was analysed by SDS–PAGE and a single protein band at the expected molecular mass of 32.5 kDa was seen, showing that Tci-gal-1 was successfully expressed and purified as a soluble protein (Figure 1A). Mass spectrometry was performed on the purified recombinant protein to confirm its identity.

To confirm if the recombinant Tci-gal-1 was functional, lactose affinity chromatography was conducted [29]. Tci-gal-1 was shown to bind to the lactose-Sepharose beads and was eluted with lactose, indicating that Tci-gal-1 was showing lectin-binding activity (Figure 1B). It was apparent that Tci-gal-1 was seen in the wash fraction which may be due to the galectin overloading the resin binding capacity. Circular dichroism spectroscopy revealed a predominantly beta-sheet secondary structure, conforming to known spectra (Supplementary Figure S2) [30].

### 3.2. Tci-gal-1-Binding Glycoproteins

**Figure 2:**
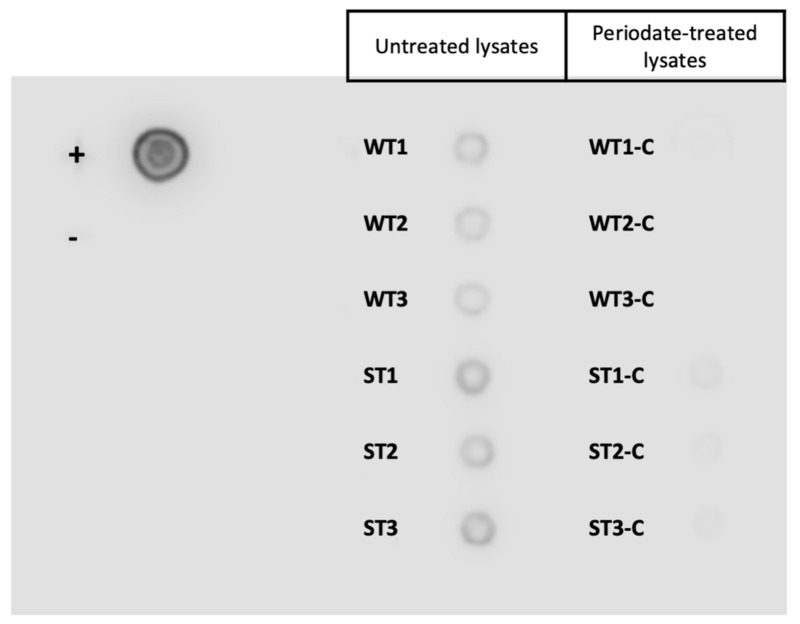
Confirmation of disruption of glycan structures by sodium periodate. ConA lectin dot blot confirming successful disruption of glycan structures after treatment of sheep abomasal whole tissue (WT) and scrape tissue (ST) with 20 mM sodium periodate. Approximately 20 µL of each sample was dotted onto a polyvinylidene difluoride (PVDF) membrane and probed with horseradish peroxidase conjugated Concanavalin A (ConA-HRP) lectin. (WT1-3) Biological replicates of abomasal whole tissue extracts. (ST1-3) Biological replicates of abomasal scrape extracts. (−C) Periodate-treated negative control samples. (+) *Fasciola hepatica* whole worm extract used as a positive control. (−) Periodate treated *Fasciola hepatica* whole worm extract used as negative control.

**Figure 3:**
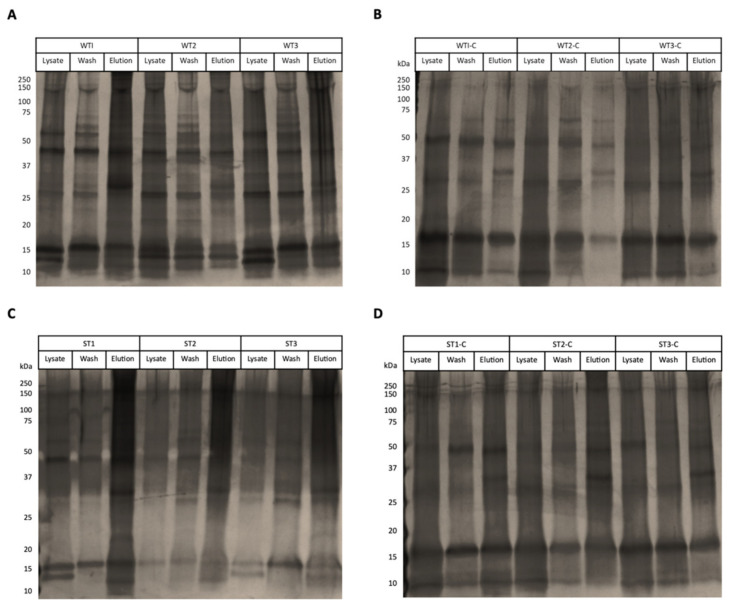
Protein profiles of abomasal whole tissue (WT) and abomasal scrape tissue (ST) extracts that bound to Tci-gal-1. Silver stained 12% (*w*/*v*) SDS-PAGE gels showing protein abomasal whole tissue and scrape tissue lysates bound to immobilised *Teladorsagia circumcincta* galectin (Tci-gal-1). (**A**) Lysate, wash and elution prepared from sheep abomasal whole tissue added to immobilised Tci-gal-1, performed in triplicate. (**B**) Lysate, wash and elution prepared from sheep abomasal whole tissue, treated with 20 mM sodium periodate, added to immobilised Tci-gal-1, performed in triplicate. (**C**) Lysate, wash and elution prepared from sheep abomasal scrape tissue added to immobilised Tci-gal-1, performed in triplicate. (**D**) Lysate, wash and elution prepared from sheep abomasal scrape tissue, treated with 20 mM sodium periodate.

The elution profile of galectin-affinity chromatography from WT and ST lysates showed minimal differences between replicates. The treatment of lysates with 20 mM sodium periodate successfully altered the glycan structures as demonstrated by the lectin dot blot (Figure 2), where the periodate-treated lysates were substantially less recognised by the lectin ConA. This suggests that the bands visualised on the silver stained SDS-PAGE showed non-specific binding of protein extracts to the resin complex in the negative controls (Figure 3). These non-specific proteins found in the periodate-treated controls were identified across the WT and ST dataset and were excluded (Supplementary Table S1).

**Figure 4:**
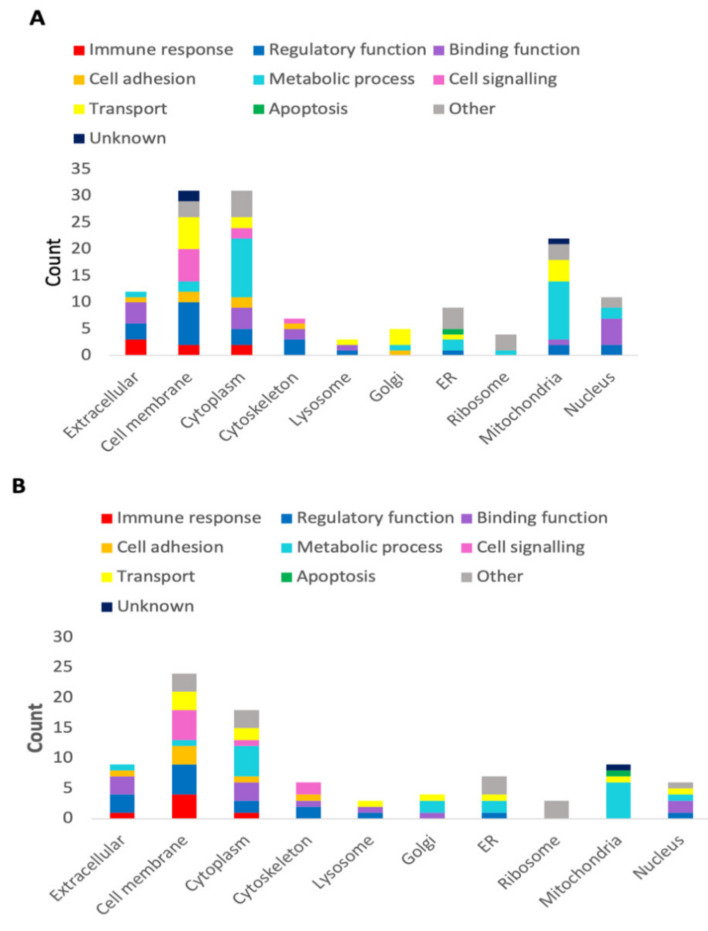
Characterisation of proteins in sheep abomasal tissue that interacted with *Teladorsagia circumcincta* galectin (Tci-gal-1). The profiles were categorised based on biological processes and cellular locations of Tci-gal-1-bound abomasal whole tissue (**A**) and Tci-gal-1-bound abomasal scrape tissue (**B**).

An analysis by MS/MS revealed a total of 990 and 821 proteins captured from the WT and ST lysates respectively, from a diversity of cellular locations and with varying functions. The WT pull-down assay identified 135 unique proteins (Figure 4A) which bound specifically to galectin conjugated Sepharose beads, while 855 proteins were identified as non-specific (Supplementary Table S2). The major cellular localisation of unique proteins was in the cell membrane and cytoplasm, with 31 proteins in each subcellular location. Transmembrane domains were identified in 27 of the 31 defined membrane proteins. The remaining proteins were found in the mitochondria (22/135), extracellular space (12/135) nucleus (11/135), endoplasmic reticulum (ER) (9/135), cytoskeleton (7/135), Golgi apparatus (5/135), ribosome (4/135) and the lysosome (3/135) (Figure 4A). Eleven of the 135 unique proteins demonstrated no predicted glycosylation sites (Supplementary Table S2). For example, the most abundant WT protein, Solute carrier family 25 member 31, had no predicted glycosylation sites and was a mitochondrial protein. The top 50 most abundant WT proteins are displayed in Table 1.

From the ST pull-down, 89 unique proteins were isolated (Supplementary Table S3), with 732 proteins identified as non-specific. Similar to the WT data, a large proportion of the identified ST proteins were derived from the cell membrane (24/89) and cytoplasm (18/89). Transmembrane domains were identified in 22 of 24 membrane proteins. This was followed by proteins in the extracellular space (9/89), mitochondria (9/89), ER (7/89), nucleus (6/89), cytoskeleton (6/89), Golgi apparatus (4/89), ribosome (3/89) and lysosome (3/89) (Figure 4B). Seven of the 89 unique proteins had no predicted glycosylation sites (Supplementary Table S2). Non-glycosylated proteins in both datasets consisted of mainly ribonuclear transport proteins. The top 50 most abundant ST proteins are displayed in Table 2.

**Figure 5:**
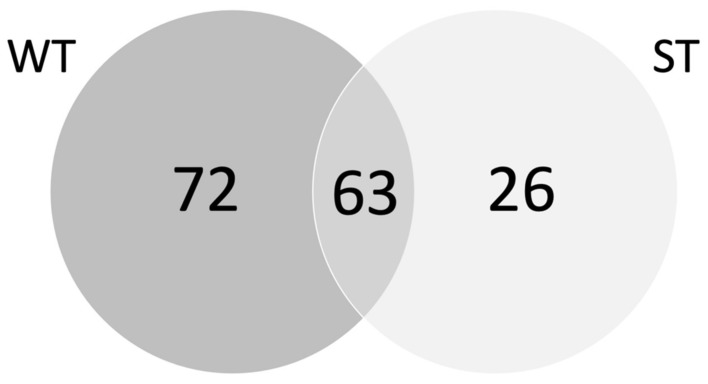
Venn diagram showing the distribution of sheep abomasal whole tissue (WT) and abomasal scrape tissue (ST) that specifically bound to *Teladorsagia circumcincta* galectin (Tci-gal-1).

The pull-down assays from both tissue types showed a large proportion of overlapping proteins, with 63 proteins observed to be present in both datasets (Figure 5). The extracellular immune protein trefoil factor 2 (TFF2) presented as the most abundant protein in ST samples and the second most abundant in WT samples, alongside other important immune proteins such as integrins, immunoglobulins, major histocompatibility complex (MHC) domains, and an angiotensin-converting enzyme (ACE). Another notable protein was the sea urchin sperm, enterokinase and agrin (SEA) domain-containing proteins which was present in both datasets and are related to mucins, along with an Fc fragment of IgG binding protein (FCGBP) in the WT sample.

## 4. Discussion

*T. circumcincta* galectin (Tci-gal-1) has the potential to modulate the host immune response [20]. However, the function of this parasite galectin remains to be elucidated. This study demonstrates that functional Tci-gal-1 can be expressed and purified from a yeast expression system, allowing for the identification of ligands from sheep abomasum whole and scrape tissue using mass spectrometry. A total of 135 proteins from abomasal tissue and 89 proteins from surface scrapes bound Tci-gal-1-conjugated resin in a carbohydrate-dependent manner. Of these proteins, 63 were present in both tissue types. The investigation of these ovine abomasal proteins focused on extracellular and membrane proteins, as parasite derived galectin is unlikely to interact with the subcellular proteins in the mitochondria, cytoskeleton, ER and ribosome during infection [24]. This study indicates that Tci-gal-1 interacts with an array of host glycoproteins, potentially including interactions important for parasite survival. However, it should be noted that the abomasum tissue was obtained from sheep killed at the local abattoir; thus, their life histories could not be obtained and therefore we do not know about previous infections or treatment for infection such drenching. Several studies have suggested the results from large experimental single infections may not reflect the same immune response as a natural infection which occurs gradually [31,32,33]. As such, we think proteins identified from tissue obtained from sheep grazed under natural conditions would be representative of what is happening at the host–parasite interface.

The membrane protein TFF2 was a highly abundant protein identified in both sample types. Trefoil factors are cysteine-rich proteins that are secreted in mucus, forming complex structures with mucins that can influence mucus viscosity [34,35]. The trefoil factor family (TFF) consists of three peptide variants: TFF1, TFF2 and TFF3 [34,36]. This family of proteins are typically expressed in response to mucosal damage [36]. Previous findings have shown that all three members of the TFF are rapidly induced upon injury, with TFF2 being upregulated as quickly as 30 min post-injury [36]. TFF2 has two trefoil domains, compared to one domain in the other variants, and is more compact in structure, which produces a higher viscosity upon interaction with mucins [34]. TFF2 may aid in nematode expulsion as shown in a study in mice infected with the hookworm *Nippostrongylus brasiliensis* which showed a higher worm burden in TFF2 deficient mice [37]. Tc-gal-1 potential aids parasite survival by reducing expulsion by mucus by interfering with TFF2’s ability to increase mucus viscosity. Mucins and TFF act in conjunction with calcium activated chloride channels (CLCA1) in the membrane to influence mucus hydration via osmotic fluid transfer, ultimately altering mucus viscosity [38].

Several immunoglobulin (Ig) domains were also identified as Tci-gal-1 interacting partners in the extracellular space, such as the FCGBP, several cluster of differentiation molecules and an immunoglobulin-like cell adhesion molecule. GI nematode infections are correlated with an increased Th2-type immune response, indicated by increased levels of Th2 cytokines, granular and globular leukocytes and parasite-specific antibodies that include IgA, IgG1 and IgE [39]. Elevated IgA levels in sheep are positively correlated with *T. circumcincta* resistance, with worm length and fecundity being significantly diminished [39,40]. IgA from the gastrointestinal tract can bind to parasites or their E/S antigens, with higher levels of parasite-specific IgA found in resistant sheep [41]. On the other hand, IgE is strongly associated with immune responses against parasitic infections including *T. circumcincta* [42]. IgE can act with larval-specific IgG1 to mediate helminth expulsion, as demonstrated in Merino lambs resistant to *H. contortus* [43]. Parasite galectins are known to bind IgE [20] and galectin may act as a molecular sponge to soak up antibodies and inhibit antibody function [20]. The identification of Ig domain-containing proteins suggest that Tci-gal-1 can interact with a range of antibodies and the Ig superfamily of proteins to regulate an environment that aids in parasite survival.

A range of surface exposed cluster of differentiation (CD) molecules were also identified in both samples. CD antigens are expressed by different subsets of cells as the cells differentiate along specific myeloid and lymphoid lineages. For example, CD47, CD54 and CD63 identified in this dataset are associated with and form complexes with integrins, affecting cell function and are essential in immune responses [44,45,46].

Several mucin-like proteins were identified in this study: a mucin 1 (MUC1)-like protein, mucin 13 (MUC13)-related protein and a mucin 18 (MUC18)-related protein. Mucus is a gel-like substance that coats the GI epithelium and can act as a physical barrier, which prevents the establishment of pathogens in the host [35,47]. Mucus possesses additional functions that include the presentation of specific ligands to trap pathogens [35], possibly preventing parasitic nematodes like *T. circumcincta* from penetrating host abomasal glands [10]. Mucus is mainly comprised of high molecular weight (>1 MDa), heavily O-glycosylated glycoproteins known as mucins [35,48,49]. Mucins can be categorised into two main subtypes: secreted mucins and membrane-bound or transmembrane mucins [35,50]. Secreted mucins are responsible for the gel-like and viscous property of mucus while transmembrane mucins have both barrier and signalling functions [47]. 

In addition to mucus proteins, SEA domains related to MUC1 and MUC13 were identified in the datasets. SEA domains are hypothesised to reduce the impacts of mechanical stress, as they are located in extracellular regions of transmembrane mucins and break at their proteolytic cleavage point upon force and reduce the effects of mechanical manipulation [51,52]. Both MUC1 and MUC13 possess a single SEA domain on the C-terminal of their mucin domains [52]. MUC1 is a major gastric transmembrane mucin with anti-inflammatory properties that is critical in the defence against enteropathogenic bacteria [47,53]. Glycosylated tandem repeats of MUC1 can extend up to 500 nm above the epithelial cell surface, forming a dense glycocalyx impermeable to bacterial pathogens [47]. MUC1 is continually internalised by clathrin-mediated endocytosis and recycled back to the cell surface, expanding its carrying capacity [53]. A clathrin adaptor, a key component of clathrin-mediated endocytosis, was identified in this study. Conversely, MUC13 is a membrane-associated sialomucin typically expressed in the intestinal tract with more pro-inflammatory characteristics [53]. The identification of both mucin-like proteins and the key SEA domain highlights a potential major role of Tci-gal-1 to alter the composition of mucus and thus facilitate parasite establishment and survival.

A melanoma cell adhesion molecule (MCAM), also known as MUC18 or CD146, was isolated from abomasal scrape samples. MUC18 is part of the immunoglobulin superfamily, consisting of five Ig domains, and is important in inflammatory responses [54]. It has been shown that Muc18 is most abundant mucin detected in gills of gilthead sea bream and gene expression of *muc18* is down regulated upon infection [55,56]. In both fish and sheep, the role of this mucin’s contribution to the stability of the mucus glycocalyx remains to be investigated. 

Integrins were also amongst the top 50 most abundant proteins across both datasets, particularly ITGA1, ITGA3 and ITGB6. These cell surface receptors are comprised of an α and β subunit, with combinations of the two subunits resulting in unique binding and signalling specificities [57]. Involved in multiple signalling pathways, these glycoproteins interact with a variety of extracellular matrix proteins such as kinases, fibronectin, collagen and other molecules to mediate activities in the extracellular matrix [58]. The interaction between Tci-gal-1 and integrins may hinder these signalling pathways to occur, impairing the host’s natural response.

While the current approach identified a range of proteins that bind to Tc-gal-1 that have a potential role in aiding the survival of the parasite, additional approaches such as co-immunoprecipitation and cross-linking can further verify if identified proteins interact with Tc-gal-1. Thus, further investigation into each identified glycoprotein is warranted to understand how this interaction impacts parasite pathogenesis. 

## 5. Conclusions

In summary, parasite galectins can perform a wide range of functions, although their role in host–parasite interactions remain to be elucidated. This study has identified glycoprotein ligands in two types of host-derived abomasal samples that bound to Tci-gal-1. In particular, Tci-gal-1 interacts with host glycoproteins such as trefoil factors, immune proteins, integrins and mucins. Strong interactions between Tci-gal-1 and mucins, in combination with other mucus-associated proteins such as trefoil factors, have the possibility to limit the efficiency of nematode expulsion by potentially altering mucus viscosity. In conclusion, this study has enhanced our understanding of the *T.* *circumcincta*–sheep interface and identified novel candidates for parasite control strategies.

## Figures and Tables

**Table 1:** Identification by mass spectroscopy of top 50 most abundant sheep abomasal whole tissue (WT) proteins eluted from a Tci-gal-1 column.

Accession Number	Gene ID	Protein Annotation	Mol. Weight (kDa)	Sequence Length	Unique Peptides	Average Abundance (Log_2_ LFQ)	Number of Predicted Glycosylation Sites		Signal Peptides	Predicted Cellular Location	Predicted Function
							N-glycan	O-glycan			
W5PCT5	SLC25A31	Solute carrier family 25 member 31	28.72	262	6	24.86	0	0	N	Mitochondria	Membrane transport
W5PLZ4	TFF2	Trefoil factor 2	14.31	131	2	24.57	0	1	Y	Extracellular	Regulatory function
W5P7F8	CD63	Tetraspanin	25.83	236	4	24.37	4	0	N	Cell membrane	Regulatory function
W5P0U4	MUC1	SEA domain-containing protein (Mucin 1 related)	58.65	588	4	23.98	5	158	Y	Cell membrane	Regulatory function
W5PBS4	LRP1	LDL receptor related protein 1	502.55	4526	14	23.56	33	122	N	Cell membrane	Regulatory function
W5QAH5	-	Transcription factor, GTP-binding domain	30.79	277	1	23.51	2	1	N	Nucleus	Other
W5QA42	MFAP4	Fibrinogen C-terminal domain-containing protein	21.42	193	2	23.38	1	0	Y	Extracellular	Metabolic function
W5PQ79	RPS15	Ribosomal protein S15	16.43	141	2	23.34	0	0	N	Ribosome	Other
W5PZK7	ACTA2	Actin alpha 2, smooth muscle	42.01	377	1	23.26	1	1	N	Cytoskeleton	Regulatory function
W5P5W1	ITGA3	Integrin subunit alpha 3	115.18	1039	10	23.09	11	4	Y	Cell membrane	Cell signalling
W5PEN0	ITGA1	Integrin subunit alpha 1	129.29	1166	9	23.09	19	15	Y	Cell membrane	Regulatory function
P00922	CA2	Carbonic anhydrase 2	29.21	260	2	23.06	0	1	N	Cell membrane	Other
W5PTZ9	LOC114116824	Histone H3	15.39	136	1	22.93	0	7	N	Nucleus	Binding function
W5P8R7	FCGBP	IgGFc-binding protein	271.26	2544	17	22.92	10	44	Y	Extracellular	Binding function
W5PNH6	FAM234A	Family with sequence similarity 234 member A-related	57.62	540	7	22.86	3	6	N	Cell membrane	Unknown
W5QBH1	CSRP1	Cysteine and glycine rich protein 1	18.72	175	2	22.59	3	7	N	Nucleus	Binding function
W5QB48	LOC101113624	ATP synthase, subunit F	10.84	93	2	22.59	0	3	N	Mitochondria	Metabolic function
W5NWN0	GSTZ1	Glutathione S-transferase zeta 1	26.7	240	4	22.54	4	1	N	Mitochondria	Metabolic function
W5PI50	GLRX	Glutaredoxin domain-containing protein	11.81	106	2	22.53	1	0	N	Cytoplasm	Regulatory function
W5NY03	CD163	CD163, scavenger receptor cysteine rich domain	122.35	1136	6	22.45	7	13	Y	Cell membrane	Cell signalling
W5Q5W2	SCARB2	CD36, scavenger receptor class B member 2	49.78	439	5	22.38	9	0	N	Lysosome	Binding function
W5QCQ4	DHX9	DExH-box helicase 9	142.13	1289	9	22.36	7	29	N	Nucleus	Regulatory function
W5Q436	SLC25A5	Mitochondrial carrier protein	29.33	263	3	22.34	1	0	N	Mitochondria	Regulatory function
W5PPJ0	STT3A	STT3 oligosaccharyltransferase complex catalytic subunit A	90.02	789	5	22.34	1	1	N	ER	Metabolic function
W5QD93	LOC101119050	Dehydrogenase/reductase SDR member 4-like	29.6	279	3	22.31	1	3	N	Mitochondria	Metabolic function
W5NTX6	RIPK1	Receptor interacting serine/threonine kinase 1	87.21	770	1	22.26	2	38	N	Cytoplasm	Regulatory function
W5PQK3	PFKL	ATP-dependent 6-phosphofructokinase	82.65	752	5	22.23	2	1	N	Cytoplasm	Metabolic function
W5Q8K4	SLC3A2	Solute carrier family 3 member 2	63.34	577	5	22.22	3	7	N	Cytoplasm	Metabolic function
W5QFP1	PABPC4	Polyadenylate-binding protein	72.31	660	2	22.19	0	8	N	Cytoplasm	Binding function
W5PQS4	PROCR	MHC class 1-like antigen recognition domain	27.09	241	3	22.18	4	1	Y	Cytoskeleton	Cell signalling
W5QJ31	MTHFD1	Methylenetetrahydrofolate dehydrogenase 1	105.89	977	6	22.17	0	4	N	Cytoplasm	Metabolic function
Q863C4	ITGB6	Integrin beta-6	85.75	787	3	22.17	7	17	Y	Cell membrane	Cell signalling
W5QFZ8	RPL13	Ribosomal protein L13	23.41	203	2	22.14	1	4	N	Ribosome	Other
W5PEE9	LAMP1	Lysosomal associated membrane protein 1	42.1	392	3	22.13	17	5	N	Lysosome	Regulatory function
W5Q9K1	QSOX1	Sulfhydryl oxidase	81.6	747	5	22.11	2	30	Y	Golgi	Metabolic function
W5NQL2	OLA1	Obg-like ATPase 1	47.25	417	6	22.09	3	2	N	Nucleus	Binding function
W5PTV7	ACE	Angiotensin-converting enzyme	138.24	1206	5	22.08	6	12	N	Extracellular	Binding function
W5P375	TCP1	T-complex 1	60.52	558	6	22.07	0	3	N	Cytoplasm	Regulatory function
W5PVM5	LAMA5	Laminin subunit alpha 5	375.79	3464	5	22.07	13	173	N	Extracellular	Regulatory function
W5QC22	CD47	CD47 molecule	32.16	291	3	22.07	5	4	N	Extracellular	Immune response
W5QCP9	COL6A3	Collagen type VI alpha 3 chain, von Willebrand factor A domain	340.43	3154	6	22	6	86	Y	Extracellular	Binding function
W5PHN8	AKR	Aldo-keto reductase	36.67	323	2	21.99	1	0	N	Cytoplasm	Metabolic function
W5P061	ABCC3	ATP binding cassette subfamily C member 3	169.72	1526	5	21.97	6	13	N	Cell membrane	Ion transport
W5Q633	BLVRA	Biliverdin reductase A	33.61	296	4	21.94	0	0	N	Cytoplasm	Metabolic function
W5PEH7	SRP68	Signal recognition particle subunit SRP68	70.9	626	6	21.9	0	19	N	Cytoplasm	Binding function
W5QEY8	GFPT1	Glutamine--fructose-6-phosphate transaminase 1	67.81	603	4	21.86	1	1	N	Cytoplasm	Metabolic function
W5Q2E5	PTPRC	Protein tyrosine phosphatase receptor type C	139.33	1235	4	21.83	19	55	N	Cell membrane	Cell signalling
W5Q496	RPL36	60S ribosomal protein L36	12.21	105	3	21.81	0	4	N	Ribosome	Metabolic function
W5P6U5	LMAN1	Lectin, mannose binding 1	52.87	465	5	21.79	0	4	N	Golgi	Protein transport
W5PK26	LASP1	LIM and SH3 protein 1	29.7	260	4	21.74	0	9	N	Cytoplasm	Binding function

**Table 2:** Identification by mass spectroscopy of top 50 most abundant sheep abomasal scrape tissue (ST) proteins eluted from a Tci-gal-1 column.

Accession Number	Gene ID	Protein Annotation	Mol. Weight (kDa)	Sequence Length	Unique Peptides	Average Abundance (Log_2_ LFQ)	Number of Predicted Glycosylation Sites		Signal Peptides	Predicted Cellular Location	Predicted Function
							N-glycan	O-glycan			
W5PLZ4	TFF2	Trefoil factor 2	14.31	131	2	24.21	0	1	Y	Extracellular	Regulatory function
W5QAH5	-	Transcription factor, GTP-binding domain	30.79	277	1	23.93	2	1	N	Nucleus	Other
W5PQ79	RPS15	Ribosomal protein S15	16.43	141	2	23.8	0	0	N	Ribosome	Other
W5P5W1	ITGA3	Integrin subunit alpha 3	115.18	1039	10	23.64	11	4	Y	Cell membrane	Cell signalling
W5QB48	LOC101113624	ATP synthase, subunit F	10.84	93	2	23	0	3	N	Mitochondria	Metabolic function
W5PBS4	LRP1	LDL receptor related protein 1	502.55	4526	14	22.89	33	122	N	Cell membrane	Regulatory function
W5PEN0	ITGA1	Integrin subunit alpha 1	129.29	1166	9	22.86	19	15	Y	Cell membrane	Regulatory function
W5P1J8	CAO	Copper amine oxidase	85.25	766	3	22.71	4	11	Y	Cytoplasm	Metabolic function
Q863C4	ITGB6	Integrin beta-6	85.75	787	3	22.58	7	17	Y	Cell membrane	Cell signalling
W5Q9K1	QSOX1	Sulfhydryl oxidase	81.6	747	5	22.46	2	30	Y	Golgi	Metabolic function
W5P5W6	NDRG1	N-myc downstream regulated 1	44.65	414	3	22.36	0	19	N	Cytoplasm	Regulatory function
W5NVK4	SPCS1	Signal peptidase complex subunit 1	18.11	163	1	22.34	1	2	N	ER	Other
W5NY03	CD163	CD163, scavenger receptor cysteine rich domain	122.35	1136	6	22.3	7	13	Y	Cell membrane	Cell signalling
W5P3H8	IGF2R	Insulin like growth factor 2 receptor, mannose-6-phosphate receptor	271.7	2463	7	22.28	16	13	N	Golgi	Binding function
W5PVR9	ERMP1	Endoplasmic reticulum metallopeptidase 1	100.02	905	4	22.28	2	5	N	ER	Metabolic function
W5QA42	MFAP4	Fibrinogen C-terminal domain-containing protein	21.42	193	2	22.21	1	0	Y	Extracellular	Metabolic function
W5QCH8	NPTN	Neuroplastin	42.43	376	6	22.21	4	3	N	Cell membrane	Cell adhesion
W5P6A5	SPINT2	Serine peptidase inhibitor, Kunitz type 2	27.96	250	2	22.17	2	9	Y	Cell membrane	Regulatory function
W5PEE9	LAMP1	Lysosomal associated membrane protein 1	42.1	392	3	22.15	17	5	N	Lysosome	Regulatory function
W5QC22	CD47	CD47 molecule	32.16	291	3	22.12	5	4	N	Cell membrane	Cell adhesion
W5NTX6	RIPK1	Receptor interacting serine/threonine kinase 1	87.21	770	1	22.1	2	38	N	Cytoplasm	Regulatory function
W5PTV7	ACE	Angiotensin-converting enzyme	138.24	1206	5	22.07	6	12	N	Extracellular	Binding function
W5P1H4	CTL4	Choline transporter-like protein 4	79.6	711	3	21.98	8	6	N	Cell membrane	Membrane transport
W5P180	MTX1	Metaxin	32.19	285	4	21.95	0	5	N	Mitochondria	Protein transport
W5Q0Z6	MCAM	Melanoma cell adhesion molecule (MUC18-related)	68.13	615	11	21.9	6	6	N	Cell membrane	Immune response
W5NYJ4	DKC1	PUA domain-containing protein	56.76	506	3	21.86	0	20	N	Nucleus	Regulatory function
W5QG70	MUC13	SEA domain-containing protein	53.93	508	2	21.79	9	62	Y	Cell membrane	Cell signalling
W5NYN6	RTN3	Reticulon	111.06	1028	1	21.78	1	151	N	ER	Other
W5P0R5	RPL21E	Ribosomal protein L21e	18.41	160	1	21.71	0	0	N	Ribosome	Other
W5Q8Y5	HDLBP	High density lipoprotein binding protein/Vigilin	141.91	1273	3	21.71	0	30	N	Nucleus	Binding function
W5PQ27	GATD3A	Glutamine Amidotransferase Like Class 1 Domain Containing 3A	28.69	274	4	21.66	1	8	Y	Mitochondria	Unknown
W5PK26	LASP1	LIM and SH3 protein 1	29.7	260	4	21.66	0	9	N	Cytoplasm	Binding function
W5Q501	GNAS	GNAS complex locus	111.42	1037	3	21.63	1	71	N	Cytoplasm	Cell signalling
W5PQS4	PROCR	MHC class 1-like antigen recognition domain	27.09	241	3	21.61	4	1	Y	Cytoskeleton	Cell signalling
W5QJ31	MTHFD1	Methylenetetrahydrofolate dehydrogenase 1	105.89	977	6	21.61	0	4	N	Cytoplasm	Metabolic function
W5PVM5	LAMA5	Laminin subunit alpha 5	375.79	3464	5	21.6	13	173	N	Extracellular	Regulatory function
W5QFP1	PABPC4	Polyadenylate-binding protein	72.31	660	2	21.6	0	8	N	Cytoplasm	Binding function
W5PD64	ARL8B	ADP ribosylation factor-like GTPase 8B	20.41	176	3	21.57	2	0	N	Lysosome	Binding function
W5PDH7	NPC1	Niemann-Pick C type protein	141.53	1275	4	21.54	14	9	Y	Lysosome	Lipid transport
W5P066	CYB5B	Cytochrome b5 type B	16.98	153	2	21.49	0	1	N	Mitochondria	Metabolic function
W5P900	RPL29	60S ribosomal protein L29	17.13	156	2	21.48	2	0	N	Ribosome	Other
W5P246	TM9SF2	Nonaspanin	76.13	666	3	21.46	0	2	Y	Cell membrane	Membrane transport
W5P1G5	ERGIC1	Endoplasmic reticulum-golgi intermediate compartment 1	32.62	290	1	21.46	1	0	N	Nucleus	ER-Golgi transport
W5P906	DPP4	Dipeptidyl peptidase 4	88.44	765	3	21.45	9	7	Y	Cell membrane	Metabolic function
W5PWS0	CD46	Membrane cofactor protein CD46	39.43	363	3	21.35	3	10	Y	Cell membrane	Immune response
W5PZG7	PECAM1	Platelet and endothelial cell adhesion molecule 1 (Ig-like)	90.96	812	3	21.35	11	2	N	Extracellular	Immune response
W5QG66	ITGB5	Integrin beta-5	88.13	802	3	21.31	5	25	N	Cell membrane	Cell signalling
W5P8N8	IDH3B	Isocitrate dehydrogenase [NAD] subunit	42.5	385	4	21.27	0	6	N	Mitochondria	Metabolic function
W5PXR3	ENPP1	Ectonucleotide pyrophosphatase/phosphodiesterase 1	94.68	823	4	21.27	6	8	N	Extracellular	Regulatory function
W5Q3J8	HLA-DRB3	MHC class II antigen DRB3	27.99	244	2	21.23	1	1	Y	Cell membrane	Immune response

## Data Availability

All data are contained within the article or supplementary material.

## Supplementary Materials

The following are available online at https://www.mdpi.com/article/10.3390/vetsci8100216/s1. Figure S1: Protein sequence of recombinant Tci-gal-1 as identified by mass spectrometry. Figure S2: Circular dichroism spectra of recombinant Tci-gal-1, Table S1: Sheep proteins that were identified in the resin control, Table S2: Identification by mass spectroscopy of sheep abomasal whole tissue (WT) proteins eluted from a Tci-gal-1 column, Table S3: Identification by mass spectroscopy of sheep abomasal scrape tissue (ST) proteins eluted from a Tci-gal-1 column.
