# Supplementary figures and images for: Teladorsagia Circumcincta Galectin-Mucosal Interactome in Sheep
Source: Vet Sci. 2021 Oct 4;8(10):216. doi: 10.3390/vetsci8100216 (PMC8540209; doi:10.3390/vetsci8100216)

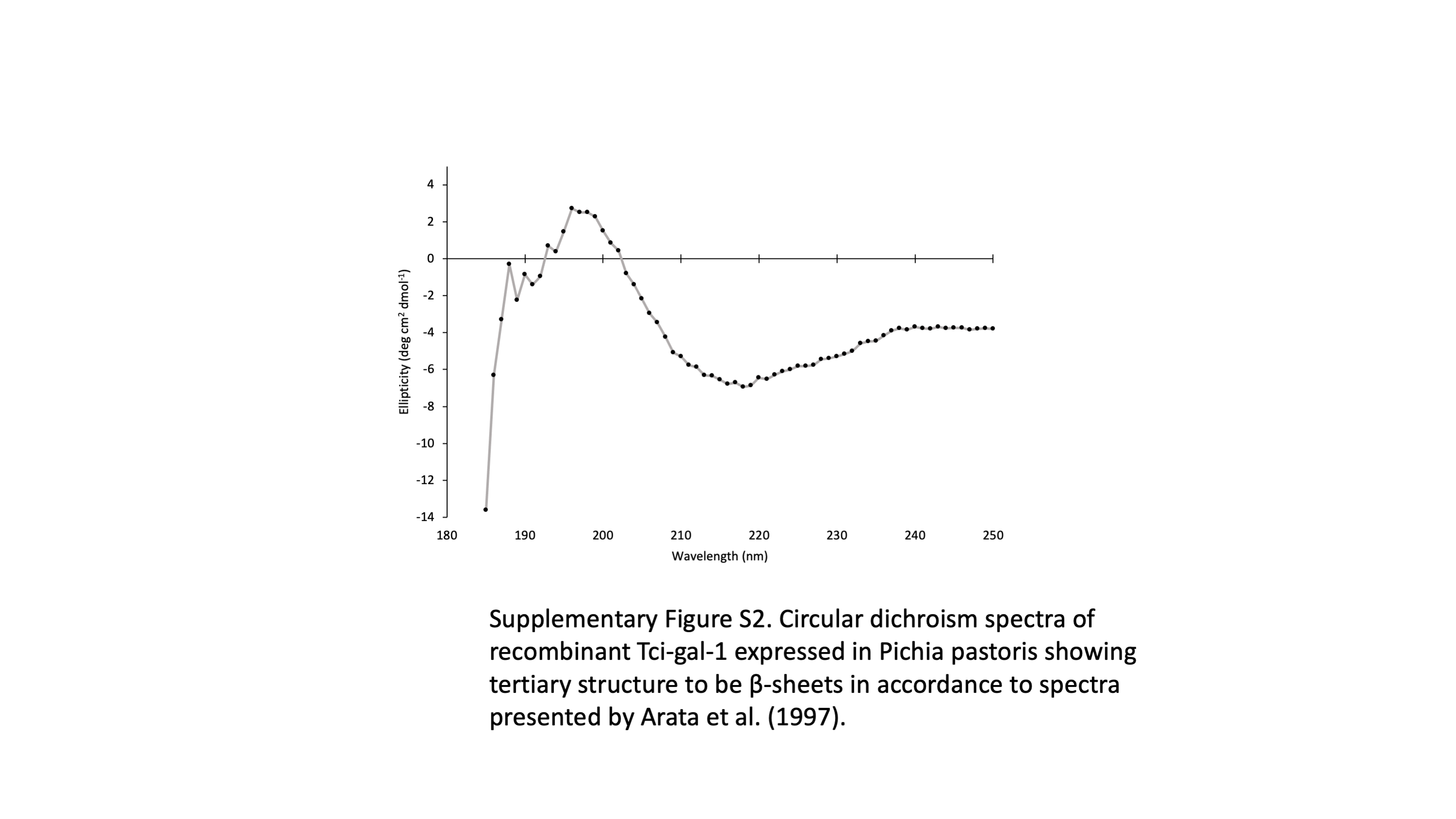

Supplement: Supplementary file 1 [file vetsci-08-00216-s001.zip › fig S2 cd.png]

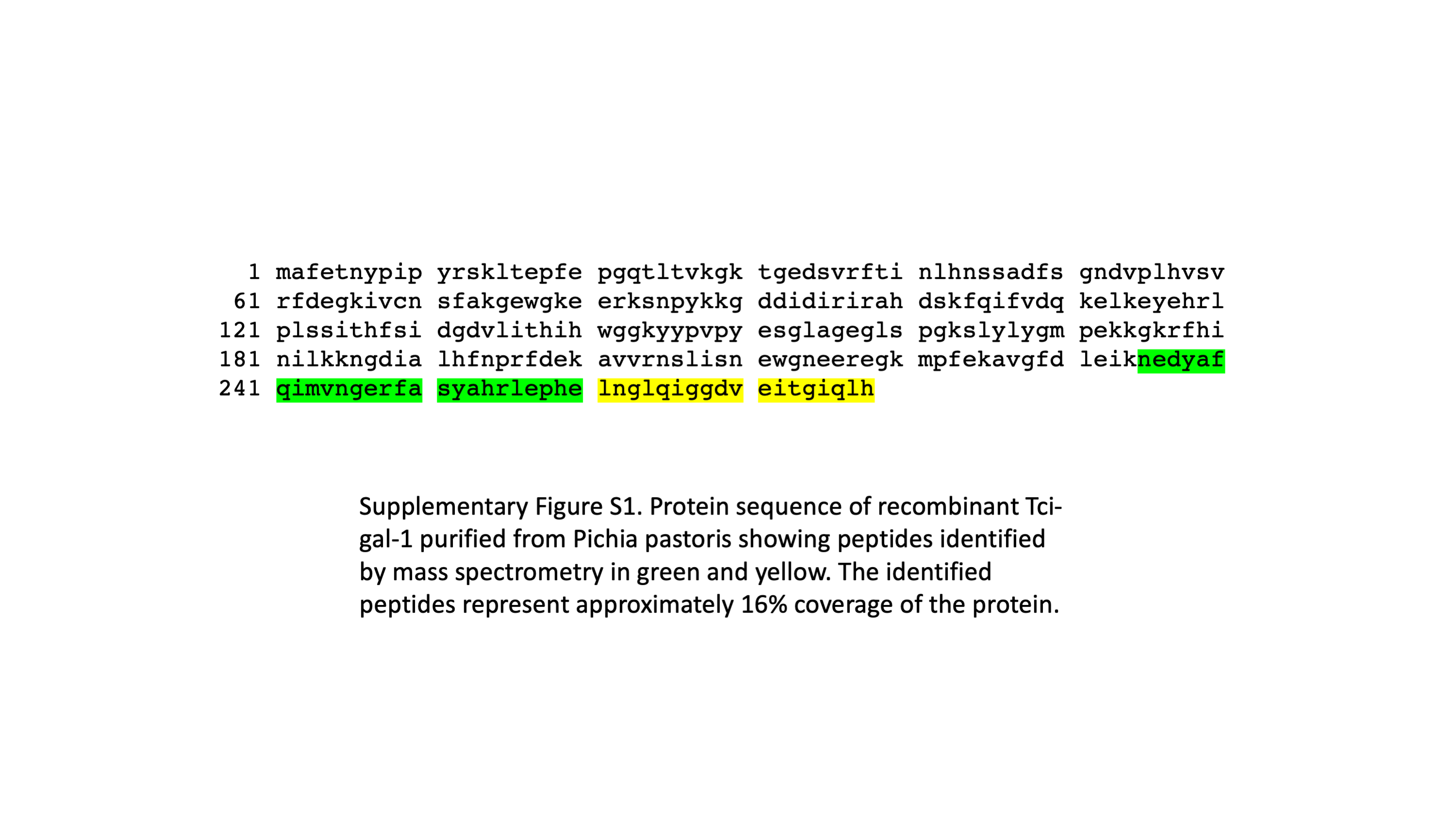

Supplement: Supplementary file 1 [file vetsci-08-00216-s001.zip › fig S1 MS.png]
